# Label-Free Multiplexed Microfluidic Analysis of Protein Interactions Based on Photonic Crystal Surface Mode Imaging

**DOI:** 10.3390/ijms24054347

**Published:** 2023-02-22

**Authors:** Galina Nifontova, Irina Petrova, Evgeniia Gerasimovich, Valery N. Konopsky, Nizar Ayadi, Cathy Charlier, Fabrice Fleury, Alexander Karaulov, Alyona Sukhanova, Igor Nabiev

**Affiliations:** 1 Laboratoire de Recherche en Nanosciences, LRN-EA4682, Structure Fédérative de Recherche Cap Santé, UFR de Pharmacie, Université de Reims Champagne-Ardenne, 51100 Reims, France; galina.nifontova@univ-reims.fr; 2 Laboratory of Nano-Bioengineering, Moscow Engineering Physics Institute, National Research Nuclear University MEPhI, 115522 Moscow, Russia; nanomedicine.mephi@gmail.com (I.P.); ewgenia-gerasimowitch@yandex.ru (E.G.); 3 Institute of Spectroscopy RAN, 108840 Moscow, Russia; konopsky@isan.troitsk.ru; 4 Nantes Université, CNRS, UMR 6286, US2B, DNA Repair Group, 44000 Nantes, France; nizar.ayadi@etu.univ-nantes.fr (N.A.); fabrice.fleury@univ-nantes.fr (F.F.); 5 Nantes Université, CNRS, UMR 6286, US2B, IMPACT Platform and SFR Bonamy, 44000 Nantes, France; cathy.charlier@univ-nantes.fr; 6 Department of Clinical Immunology and Allergology, Institute of Molecular Medicine, Sechenov First Moscow State Medical University (Sechenov University), 119146 Moscow, Russia; drkaraulov@mail.ru

**Keywords:** photonic crystal surface mode imaging, label-free biosensing, protein array, multiplexed detection, immunoassay

## Abstract

High-throughput protein assays are crucial for modern diagnostics, drug discovery, proteomics, and other fields of biology and medicine. It allows simultaneous detection of hundreds of analytes and miniaturization of both fabrication and analytical procedures. Photonic crystal surface mode (PC SM) imaging is an effective alternative to surface plasmon resonance (SPR) imaging used in conventional gold-coated, label-free biosensors. PC SM imaging is advantageous as a quick, label-free, and reproducible technique for multiplexed analysis of biomolecular interactions. PC SM sensors are characterized by a longer signal propagation at the cost of a lower spatial resolution, which makes them more sensitive than classical SPR imaging sensors. We describe an approach for designing label-free protein biosensing assays employing PC SM imaging in the microfluidic mode. Label-free, real-time detection of PC SM imaging biosensors using two-dimensional imaging of binding events has been designed to study arrays of model proteins (antibodies, immunoglobulin G-binding proteins, serum proteins, and DNA repair proteins) at 96 points prepared by automated spotting. The data prove feasibility of simultaneous PC SM imaging of multiple protein interactions. The results pave the way to further develop PC SM imaging as an advanced label-free microfluidic assay for the multiplexed detection of protein interactions.

## 1. Introduction

High-throughput protein analysis has emerged as an essential part of drug discovery and of the diagnosis and molecular profiling of various diseases, including cancer, autoimmune disorders, and infections [1,2,3]. The use of protein arrays and chip-based analysis ensures quick, multiplex, and sensitive analysis of target proteins, and has been widely employed for the screening and monitoring of protein–protein interactions and for assessing their kinetics [4,5,6,7]. The advantages of protein chip-based assays also include the use of small analyte volumes, as well as miniaturization and automatization of the analytical procedure.

The detection techniques employed in protein chip analysis can be categorized into labeling detection techniques (e.g., fluorescence analysis) and label-free detection techniques. Label-free approaches based on the estimation of refractive index changes at the substrate–liquid interface has been widely used along with traditional labeling-based techniques for the detection of analytes [8,9]. The obvious benefit of label-free arrays is reduced time and material cost of analysis compared with traditional labeling approaches using fluorescence, Förster resonance energy transfer (FRET), absorbance, or chemiluminescence [10].

The surface plasmon resonance (SPR) technique is one of the most frequently used label-free optical methods. It is based on the excitation of surface plasmon polaritons along the metal–dielectric interface by incident light [11,12]. The SPR-based techniques detect local changes in the refractive index that occur at the surface of the thin gold coating of the sensor due to the interaction between the analyte and the immobilized capture molecules [13,14]. In addition to the conventional SPR, its modification known as SPR imaging has been developed to ensure the simultaneous detection of multiple adsorption interactions by real-time imaging with the use of a patterned two-dimensional (2D) array on the gold film surface [15,16]. The interaction between the protein analytes and capture molecules deposited on the sensor surface is recorded by a CCD camera and represented as an image where the intensity of binding is shown in a color scale [17,18]. However, SPR decay on the metal film has been shown to considerably limit the sensitivity of SPR sensors [19]. In addition, SPR imaging suffers from an order of magnitude poorer resolution than the classical SPR technique (10^−6^ versus 10^−7^ RU), which calls for the engineering of more efficient analogues of sensors coated with metal (gold/silver) films [17].

Devices employing the photonic crystal (PC) surface mode (SM) have recently emerged as an effective alternative to the noble metal-coated biosensors employing the SPR phenomenon [20]. PCs are periodic multilayer structures with a periodically changing refractive index in the optical spectrum in one, two, or three dimensions [21]. The optical field near the PC surface is strongly confined due to the photonic band gap in the multilayer structure on the internal side of the outer PC surface and the total internal reflection on the external side, which is in contact with the liquid. PCs operating at any wavelength can be fabricated, whereas the SPR operation spectrum is limited to the red and near-infrared ranges, where gold has a small imaginary part of the permittivity. PC surface modes (SMs) do not suffer from metal damping. The length of PC SM propagation is one to two orders of magnitude longer than the SP propagation length (due to the absence of dissipation of optical energy in the metal), which significantly enhances the sensitivity of the PC SM devices [22,23]. PCs enable simultaneous detection of two surface modes with different penetration depths, which allows for separate recording of signals both from the surface and from the bulk liquid. Thus, the adlayer changes can be differentiated from the changes in the refractive index due to the temperature or chemical composition of the liquid sample [24,25].

To monitor multiple interactions, an imaging PC SM sensor has been designed [26]. An increase or decrease in the adsorption layer thickness caused a shift in the PC SM peak to longer or shorter wavelengths, respectively, thus increasing or decreasing the green component (G) and simultaneously decreasing or increasing the blue component (B) of the color pixel response of the color camera mounted above the PC surface to detect binding events.

The normalized differential value (B − G)/(B + G) can be used as a measure of the PC SM resonance wavelength shift free from intensity fluctuations. In contrast, in SPR imaging biosensors, fluctuations of the intensity of the light source are directly transmitted into a useful signal. The reason is that, to obtain a 2D image of adlayer thickness variations in SPR biosensors, the laser incidence angle is tuned to the slope of the resonance dip in the SPR reflection curve.

PCs are chemically and physically stable, which offers wide possibilities for their use and adaptation for biosensing approaches [27]. The same PC chip can be used many times because the thick final SiO_2_ layer can be effectively cleaned by some active treatment (e.g., in a plasma or UV cleaner). The use of PCs is also an advantage because of the highly developed silane chemistry allowing the conjugation of biological molecules to the surface [28]. Therefore, the PC surface can be modified for coupling of capture molecules using aminosilanization [24,25]. Additional treatment of the surface with glutaraldehyde or carbodiimide cross-linkers enables effective immobilization of aptamers and capture molecules [24,29,30]. PC-based sensors have been reported as an array platform for multiplexed detection of cancer biomarkers at concentrations of several picograms or even less than a picogram per milliliter [22,31]. Thus, the sensitivity of PC SM biosensors can surpass the sensitivity of SPR metal-coated biosensors [32].

This study was aimed at designing a label-free assay employing PC SM imaging for the multiplexed detection of protein–protein interactions in the lateral-flow microfluidic format. Here, we describe an approach to PC surface functionalization; the design of a chip containing 13 model proteins/peptides, including secondary and primary antibodies (Abs), His-tagged (RAD51) and phosphorylated proteins (phosphorylated bovine serum albumin (BSA)), and phosphorylated and non-phosphorylated peptide fragments of RAD51 and BSA; and PC SM imaging analysis of interactions between Ab analytes and spotted proteins. The results of label-free detection of interactions of Abs, proteins, and peptides based on PC SM imaging performed in the microfluidic format are presented.

The difference of the present work from previously published data [22] is in the (1) simultaneous measurement of molecular interactions of a significant number of very different proteins deposited on thePC, and (2) the first-time evaluation of the optimal amount of protein for depositing that provides a clear signal increase when an analyte solution is introduced into the microfluidic cell. Thus, the results are not just a demonstration of multiparameter detection capability using the described system, but a real proof-of-concept paving the way to future use of PC SM imaging systems as advanced tools for label-free detection of protein interactions applicable for high-throughput screening in diagnostics, drug discovery, and enzymatic kinetics applications.

## 2. Results and Discussion

### 2.1. Chip Biofunctionalization and Possible Protein Interactions

**Figure 1:**
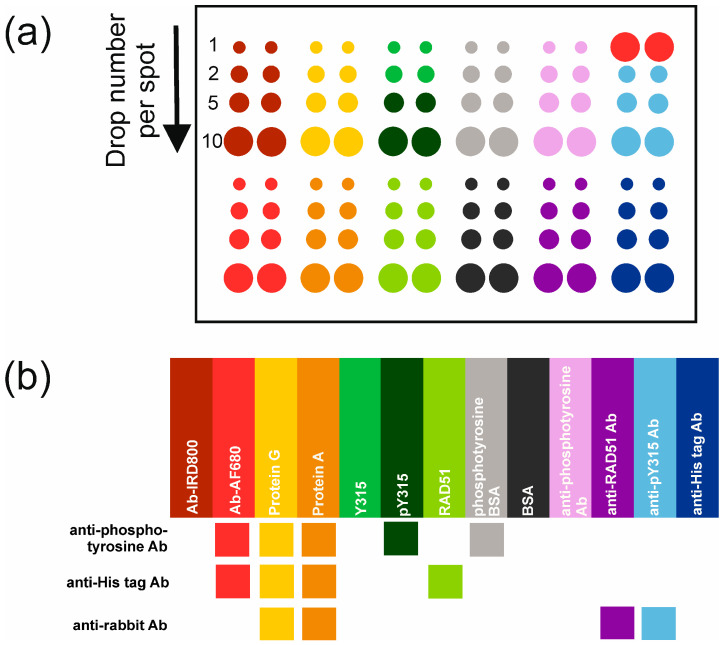
A schematic diagram of the photonic crystal chip with proteins and peptides deposited and possible interactions between the deposited spots and injected analytes. (**a**) The layout of spotting of model proteins and peptides. (**b**) The possible interactions between the proteins. The arrow in panel A indicates an increasing number of drops per spot. Abbreviations: Ab, antibody; Ab-AF680, secondary antibody conjugated with Alexa Fluor 680; Ab-IRD800, secondary antibody conjugated with IRDye 800CW; Y315, Y315 peptide; pY315, phosphorylated Y315 peptide; RAD51, DNA repair protein; phosphotyrosine BSA, bovine serum albumin phosphorylated at tyrosine residues; BSA, bovine serum albumin; anti-phosphotyrosine Ab, antibody for the detection of phosphorylated proteins; anti-RAD51 Ab, antibody for the detection of the DNA repair protein; anti-pY315 Ab, antibody for the detection of the phosphorylated Y315 peptide; anti-His tag Ab, anti-polyhistidine antibody.

The efficiency of silicon-based sensors, particularly PCs, depends on the surface chemistry approach used for their functionalization and on the protein surface layer density, protein pI, hydrophobicity, conformation, etc. [33,34]. Before the spotting of proteins and peptides, the PC surface was modified by APTES self-assembly and subsequently activated with a homo-bifunctional cross-linker, e.g., glutaraldehyde, to enable effective coupling [35,36]. After modification of the aminosilanized surface with glutaraldehyde, an intermediary Schiff base forms which enhances alkoxysilane–glutaraldehyde crosslinking and ensures immobilization of proteins through linking their primary amine residues [37]. Thirteen different proteins and peptides were explored as capture molecules containing the recognition units of the sensor and were spotted on the surface of the PC SM chip according to the scheme presented in Figure 1. The spotted proteins represented Abs, immunoglobulin G-binding proteins, primary Abs, peptides, proteins, as well as phosphorylated proteins and peptides (detailed description of the proteins immobilized at the sensor surface is presented in Table S1, Supplementary Materials). RAD51 is the key protein of homologous recombination DNA repair, a mechanism of faithful repair of double-strand breaks (DSBs) involving the RAD51 foci. RAD51 activity is regulated by post-translational modifications, such as phosphorylation of tyrosine at position 315 (Y315). We have previously demonstrated that peptide phosphorylation at Y315 inhibits the RAD51 focus formation and homeologous recombination repair (HomeoRR) in leukemia cells [38]. Therefore, RAD51 and phosphorylated and non-phosphorylated Y315 peptides are the major markers of DNA damage response, and we used them as recognition units of the biosensor. In addition, RAD51 was histidine-tagged to make it detectable by anti-histidine Abs, and its phosphorylated form could be recognized by anti-pY315 rabbit Abs purified as described in the previous study [39] or by anti-phosphotyrosine mouse Abs. Commercial BSA was phosphorylated at multiple tyrosine residues by a chemical method, and this phosphorylated form was revealed using the same anti-phosphotyrosine mouse Abs. Thus, these phosphorylated and histidine-tagged proteins are also potential molecular targets to be used as the recognition part of the biosensor engineered. Immunoglobulin G-binding proteins (proteins A and G) were used for the preliminary monitoring of coupling of the Ab analytes with the sensor surface. Conjugates of secondary Abs with IRD800 (Ab-IRD800) and Alexa Fluor 680 (Ab-AF680) were spotted to verify the efficiency of spotting orientation of the sensor before its mounting onto the prism. The possibility of cross-reactive coupling of Ab analytes to the secondary Ab spots was also analyzed. BSA served as a reference protein to estimate nonspecific binding of the Ab analytes. The quantity of the proteins immobilized varied in different spots. One to ten drops with the mean volume of 0.4 nL were applied onto the chip surface (Figure 1a). The quantities of the proteins applied are presented in Table S1, Supplementary Materials. The spots were duplicated to ensure the reliability of the data. A set of unlabeled Ab analytes (anti-phosphotyrosine, anti-His tag, and anti-rabbit Abs) was used to monitor specific interactions with the target protein and the peptide spotted (Table S2, Supplementary Materials). The possible interactions between Ab analytes and proteins/peptides applied are presented in Figure 1b.

### 2.2. Photonic Crystal Surface Mode Imaging

**Figure 2:**
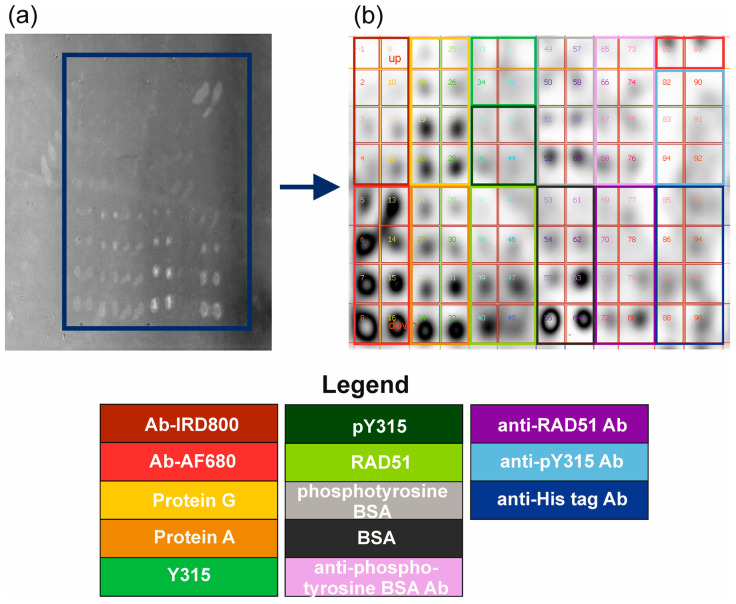
The overview of the chip surface with applied proteins and peptides. (**a**) A black-and-white 2D image of the chip placed in front of the CCD camera before antibody running. (**b**) A layout consisting of 96 regions of interest (ROIs) corresponding to the deposited spot duplicates. The protein spotting pattern corresponds to the scheme shown in Figure 1. Abbreviations: Ab, antibody; Ab-AF680, secondary antibody conjugated with Alexa Fluor 680; Ab-IRD800, secondary antibody conjugated with IRDye 800CW; Y315, Y315 peptide; pY315, phosphorylated Y315 peptide; RAD51, DNA repair protein; phosphotyrosine-BSA, bovine serum albumin phosphorylated at tyrosine residues; BSA, bovine serum albumin; anti-phosphotyrosine Ab, antibody for the detection of phosphorylated proteins; anti-RAD51 Ab, antibody for the detection of the DNA repair protein; anti-pY315 Ab, antibody for the detection of the phosphorylated Y315 peptide; anti-His tag Ab, anti-polyhistidine antibody.

The PC SM signal was detected as a change in the adlayer thickness (da, nm) simultaneously over 96 spots corresponding to 96 ROIs mapped as shown in Figure 2. The protein spots on the surface of the chip were visible immediately after placing the PC SM chip in front of the camera of the PC SM imaging device. Figure 2a represents the image of the chip before running the Ab analytes through the flow cell. The ROIs corresponding to the groups of the spotted proteins and peptides were selected to monitor Ab analyte binding (Figure 2b). The images are compressed horizontally at a ratio of about 2:1 because they appear on the camera after reflection inside the prism at the angle of 65.18°. The brightness of the spots in the black-and-white image (Figure 2a) corresponds to the amount of protein deposited in the given region of the PC chip surface.

Initially, the running buffer (0.01 M PBS, pH 7.4) was pumped through the microfluidic cell until signal equilibration. Then, the Ab analytes were injected subsequently until signal stabilization (for ~390 s). After the running of each analyte, 0.01 M PBS (pH 7.4) was injected (for ~180 s) until signal equilibration to remove unbound antibody molecules.

**Figure 3:**
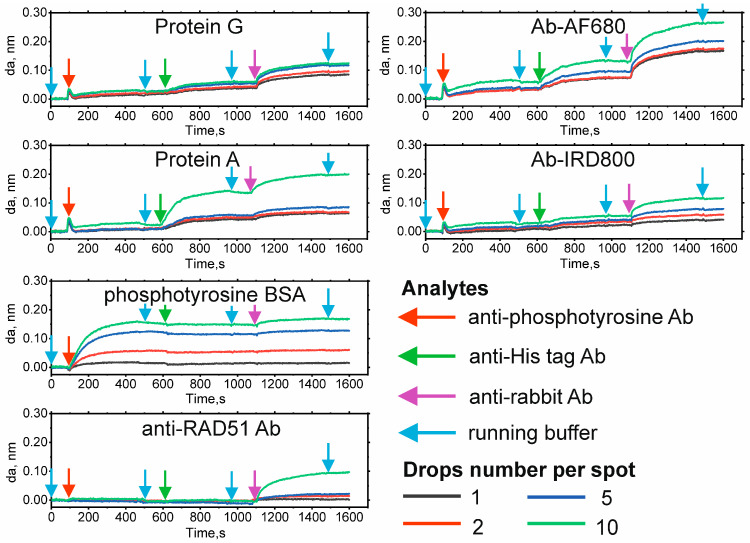
The sensorgrams of interaction of proteins, peptides, and injected antibody analytes in the selected spots. The start of the injection of each antibody analyte run through the microfluidic cell is indicated with an arrow. Each resultant sensorgram is an average of two sensorgrams recorded in duplicate. Abbreviations: da, adlayer thickness; Ab, antibody; Ab-AF680, secondary antibody conjugated with Alexa Fluor 680; Ab-IRD800, secondary antibody conjugated with IRDye 800CW; phosphotyrosine-BSA, bovine serum albumin phosphorylated on tyrosine residues; anti-RAD51 Ab, antibody for the detection of the DNA repair protein; anti-phosphotyrosine Ab, antibody for the detection phosphorylated protein and peptides; anti-His tag Ab, anti-polyhistidine antibody; anti-rabbit Ab, antibody for the detection of rabbit antibodies.

The resultant sensorgrams are shown in Figure 3 and in Figure S1 (Supplementary Materials). The interaction of proteins/peptides immobilized at the chip surface and Ab analytes also resulted in an elevated brightness of the spots, as shown in Figure S2 (Supplementary Materials).

For the evaluation of the effect of the possible nonspecific interactions of the analytes, the surface of the chip was preliminarily blocked with BSA. Additionally, the curves of the variations of adlayer thickness over control spots (BSA) monitored over time were used as reference sensorgrams. Considerable changes in the adlayer thickness at the chip surface after injection of each protein analyte distinguishable from the reference BSA spots were detected in the spots of immunoglobulin G-binding proteins A and G, as well as the Ab-AF680 conjugate.

The spots of proteins A and G exhibited different capacities of interaction with all Ab analytes. The increase in adlayer thickness over protein G spots was smaller than that over protein A ones due to smaller quantities of protein G (Table S1, Supplementary Materials). Moreover, despite the high affinity for immunoglobulins G from most species, a protein G molecule contains only two domains for binding of Fc fragments of Ab molecules and, hence, can immobilize only two Ab molecules. Protein A, on the other hand, contains five domains for binding of Fc fragments of Abs; i.e., protein A spots have a larger capacity for interacting with all the Ab analytes despite the lower affinity for immunoglobulin G molecules compared to protein G [40,41].

The monospecific interaction of proteins with a single Ab analyte was also demonstrated at the spots of anti-RAD51 Ab and phosphotyrosine-BSA after running anti-rabbit Ab and anti-phosphotyrosine Ab, respectively. In the case of the interaction of anti-phosphotyrosine Ab analyte with the other phosphorylated moiety (pY315), the binding of the Ab was negligible, which may have been caused by its insufficient quantity and the lower degree of phosphorylation of the ligand. The sensorgrams recorded over anti-RAD51 rabbit Ab spots demonstrated no interaction of the injected anti-phosphotyrosine and anti-His tag Abs, and specific interaction was observed after running anti-rabbit Ab.

In the case of the secondary Ab conjugated with AF680, specific interactions with all Ab analyte types were recorded, as it is shown in the sensorgram. However, nonspecific interaction due to Ab cross-reactivity was observed at the spots of the Ab-IRD800 conjugate. The interactions in the case of spotted pospho-Y315 and Y315 peptides, RAD51, anti-phospho-Y315 Ab, anti-His tag Ab, and anti-phosphotyrosine Ab were found to be practically negligible because the sensorgrams showed no dramatic increase in the adlayer thickness during analyte running (Figure S1, Supplementary Materials). To assess the amounts of proteins and peptides necessary for a significant response, most of them were placed onto the surface in four quantities (1, 2, 5, and 10 drops). Because the samples of different proteins had different concentrations, the actual range of the protein/peptide amounts varied from 0.004 ng to 20 ng throughout the chip (Table S1, Supplementary Materials). Higher mean values of the maximum gain of the adlayer thickness in the selected spots after running all the Ab analytes were observed upon an increase in the quantities of the proteins/peptides per spot (number of drops spotted) (Table S3, Supplementary Materials). Significant differences in the changes in the mean values of the maximum adlayer thickness in the spots studied from those of the reference BSA spots (*p* < 0.05 according to Student’s *t* test) were found in the case of immunoglobulin G-binding proteins (A and G), phosphotyrosine-BSA, Ab-AF680 conjugate, and Ab-IRD800 conjugate spots. Spots containing small amounts of anti-Rad51 Ab, corresponding to 1, 2, and 5 drops/spot, exhibited no distinct changes in the mean value of the maximum gain of adlayer thickness compared to the reference BSA spots, which may have been due to the insufficient quantity of the ligand deposited.

However, an increase in the number of anti-RAD51 Ab drops to 10 drops/spot ensured a significant increase in the signal due to the binding of the Ab analyte, which indicates that the optimal quantity range for this ligand starts from 0.5 ng (Table S1, Supplementary Materials). The mean da values for anti-phosphotyrosine Ab, phospho-Y315 peptide, Y315 peptide, RAD51, anti-phospho-Y315 Ab, and anti-His tag Ab did not differ significantly from that for the reference BSA spots (*p* > 0.05 according to Student’s *t* test), which demonstrates weak interactions of the injected analytes with the ligands spotted (Table S1, Supplementary Materials) and, hence, specificity of the observed interactions between the injected analytes and the deposited proteins/peptides.

However, to discriminate between the observed interactions and non-interactions, we should consider not only the noise value, but also the baseline drift. The sensorgram for BSA spots demonstrated a drift over time by 0.013 signal units. Thus, the experimentally observed interactions caused quantitative rises of more than 0.02 signal units. The limit of detection for mass per area in a single spot of the biosensor is determined by the baseline noise, which is 9 × 10^−4^ nm in the presented kinetic curves. This corresponds to an adlayer thickness noise of 3.2 pm. This thickness noise corresponds to 3.2 pg/mm^2^ in terms of surface mass density. This is consistent with previous data, because the EVA 3.0 device has been reported [26] to have a noise value of 3.5 pg/mm^2^. The smallest spots for which a reliable PC SM image signal was detected had an approximate area of 0.04 mm^2^ (1 drop/spot). This is almost the limit of spatial resolution of the EVA 3.0 biosensor (see the Experimental Section). For the spot size of 0.04 mm^2^, this gives a noise value of 128 fg for a single spot. Considering the limit of detection to be thrice the noise value, about 350 fg of the analyte should be immobilized in one spot to ensure Ab analyte detection. According to the published data, the limit of detection of the designed PC SM imaging sensor provides a better sensitivity compared to the established limit of detection of the classical SPR imaging approach, which is between several picograms and several nanograms of protein (Ab) analytes per milliliter [42,43]. However, the sensitivity of the approach developed is comparable with that of complex and sophisticated plasmon-nanoparticle-enhanced SPR-immunoassay, providing the limit of detection of protein analytes within the range from several femtograms to several picograms per milliliter, depending on the density of capture molecules at the sensor surface and the affinity of the Abs used [44,45]. Thus, the PC SM imaging has been demonstrated to be a promising optical label-free technique allowing the detection of ultrasmall protein quantities within the femtogram range and real-time monitoring of protein interactions. For comparison, the other label-free sensing approaches such as quartz crystal microbalance (QCM) based on chemical coating of the surface, electrochemical sensors, and field-effect transistors (FET) detect protein analytes in the nanogram to picogram range [46,47,48,49,50,51]. However, an alternative, more sensitive label-free biosensor array based on electrolyte-gated organic thin-film transistors for multiplexed detection of proteins with zeptomolar sensitivity has been recently reported [52].

## 3. Materials and Methods

### 3.1. Materials

Glutaraldehyde and 3-(aminopropyl)triethoxysilane (APTES) were purchased from Merck Group, Saint-Quentin-Fallavier, France. Absolute ethanol and absolute acetone of analytical grades were purchased from Acros Organics (through Fisher Scientific France, Illkirch, France). The IRDye^®®^ 800CW conjugate of goat anti-rabbit immunoglobulin G secondary Ab (926-32211) was purchased from Li-Cor Biosciences, Bad Homburg vor der Höhe, Germany. The goat anti-mouse immunoglobulin G (H + L) highly cross-adsorbed secondary Ab conjugated with Alexa Fluor 680 (A-21058), immunoglobulin G-binding proteins, such as protein A (21184) and protein G (21193), and BSA standard Pierce™ (23210) were obtained from Thermo Fisher Scientific, Illkirch, France.

Histidine-tagged RAD51 was prepared and purified as described in our previous study [53]. Peptides (13 amino acid residues in length) around the Y315 region of RAD51 were synthetized in the phosphorylated (pY315) or non-phosphorylated (Y315) form by Genecust Company, Boynes, France. BSA phosphorylated at tyrosine residues (phosphotyrosine-BSA, P3967) and polyclonal anti-RAD51 rabbit Ab (ABE257) were obtained from Merck Group, Saint-Quentin-Fallavier, France. Monoclonal anti-His tag Ab (H1029) and polyclonal anti-rabbit Ab were purchased from Thermo Fisher Scientific, Illkirch, France. Anti-phosphotyrosine mouse monoclonal Ab (9411S) was from Cell Signaling Technology, Ozyme, Saint-Cyr-L’École, France. Anti-phospho-Y315 rabbit antibody was purified as described previously [39]. Detailed information about the proteins used is presented in Tables S1 and S2 in the Supplementary Materials.

All other reagents used were of analytical grade. Buffer solutions were prepared using Milli-Q water (18.2 mΩ·cm) obtained by means of a Direct-Q water purification system (Merck Group, Saint-Quentin-Fallavier, France) and additionally filtered through sterile Millex-GV filters with a pore size of 0.22 μm (Merck Group, Saint-Quentin-Fallavier, France).

### 3.2. Photonic Crystal Chip Fabrication

The structure of 1D PC used for protein microarray engineering was the following: planar BK-7 glass substrate/TiO_2_(SiO_2_/TiO_2_)_5_SiO_2_/water, where the thickness of the TiO_2_ layer was 64.0 nm, the thickness of the intermediate SiO_2_ layer was 214.3 nm, and the final SiO_2_ layer was applied at the thickness of 298.8 nm. The substrate layering was performed using a SYRUSpro 710 optical vacuum coater (Buhler Leybold Optics, Alzenau, Germany) via electron-beam evaporation and plasma ion-assisted deposition. The refractive indices of the substrate and the SiO_2_ and TiO_2_ layers at *λ* = 500 nm were n_0_ = 1.52, n_1_ = 1.48, and n_2_ = 2.27, respectively.

The multilayered PC structure supports a *p*-polarized PC surface mode at *λ*= 500 nm with an effective refractive index (RI) = *n*_0_sin(*θ*_0_) = 1.38, corresponding to the in-prism excitation angle *θ*_0_ = 65° [22,54].

### 3.3. Chip Surface Modification

Before use, the multilayered PC chips were pre-cleaned by incubation and sonication in absolute ethanol and then in absolute acetone. After that, each side of the chip surface was cleaned using an UV-purifying device for 45 min and then incubated in 1% APTES in acetone for 16 h to coat the whole surface with primary amine groups. After aminosilanization, the PC chip surface was modified for subsequent protein conjugation using 2.5% glutaraldehyde as described earlier [24]. The resultant chips with a pre-activated surface were stored in 0.02 M sodium phosphate buffer, pH 7.4.

### 3.4. Protein Spotting and Chip Biofunctionalization

The solutions of proteins and peptides were prepared at concentrations of 5–5000 µg/mL in 0.01 M phosphate-buffered saline (PBS), pH 7.4, in a 384-well microplate (Porvair Sciences, Shepperton, UK) as shown in Table S1, Supplementary Materials. The spotting of the proteins and peptides was performed using a sciFLEXARRAYER S3 with a Piezo Electric Dispenser (Scienion, Berlin, Germany). One to ten 0.4 nL drops of each sample were applied onto a 150 mm^2^ area of the surface of the pre-functionalized PC chips in two replicates. The dropping was performed through a capillary and a controlled live stream camera. The average distance between the spots was 1.3 mm. The spotting was performed in a chamber at 25 °C and a humidity of 60%. Afterwards, the chips were dried to prevent smearing of the spotted capture molecules. Before use, the PC chips with spotted proteins were incubated in a 2% BSA solution to block the pre-activated protein-free surface. The prepared chips were stored at 4 °C until use.

### 3.5. Photonic Crystal Surface Mode Imaging

**Scheme 1:**
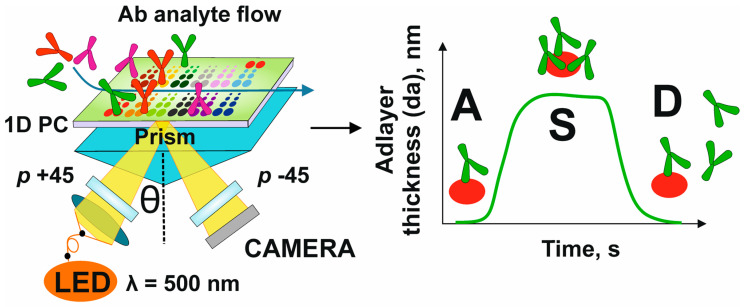
The concept of the label-free microfluidic sensor of multiple protein interactions based on photonic crystal surface mode imaging. Abbreviations: 1D PC, one-dimensional photonic crystal; Ab, antibody; p, polarizer; A, association of an antibody analyte to a protein spot; S, saturation of a protein spot with an antibody analyte; D, dissociation of an antibody–protein complex.

PC SM imaging experiments were performed using the adapted EVA 3.0 microfluidic system described earlier [22,23,26]. Briefly, a parallel light beam with a polarization of +45° to the plane of incidence illuminated a one-dimensional PC chip through a prism (Kretschmann-like configuration). After reflection, the light beam passed through the second polarizer, which is at −45° to the plane of incidence, and is recorded by a color camera. The 1D PC chip supported a p-polarized PC SM at 500 nm with an effective refractive index of 1.38, which corresponded to an in-prism excitation angle of 65°. This excitation caused a phase shift of the surface wave that re-radiated back to the prism. Therefore, the resulting polarization of the reflected beam was rotated and, as a result, the light at the excitation wavelength passed through the crossed polarizers. The PC SM excitation wavelength was 500 nm. The PC excitation wavelength corresponded to the intermediate position between the maxima of blue (~460 nm) and green (~545 nm) pixels. The excitation of the PC at this wavelength resulted in a PC resonance peak at 500 nm in the reflected LED light. The shifts of the position of the PC resonance peak due to the binding interactions at the sensor surface were calculated by analyzing both pixels intensities within the selected regions of interest (ROI) [26]. The spatial resolution of the device was 100 µm. For PC SM imaging, solutions of Ab analytes (anti-phosphotyrosine Ab, anti-His tag Ab, and anti-rabbit Ab) in 0.01 M PBS, pH 7.4 (Table S2, Supplementary Materials), were run through the microfluidic cell at a rate of 30 µL/min. A solution of 0.01 M PBS, pH 7.4, was also used as a running buffer between injections of different Abs. To monitor interactions, the spot layout of the image of the chip mounted in front of the camera was prepared. Ninety-six areas of interest corresponding to the protein/peptide spots applied were selected, and 96 parallel signals were recorded and monitored over time. The design principle of the sensor is presented in Scheme 1.

### 3.6. Data Analysis

Primary processing and statistical analysis of the imaging data was performed using the Origin Pro version 2018 software (OriginLab Corporation, Northampton, MA, USA). The results are presented as the means and standard deviations for three independent experiments if not indicated otherwise.

## 4. Conclusions

We have demonstrated that the PC SM imaging approach can be successfully used for the multiplexed, label-free analysis of multiple interactions of proteins and peptides. Combined surface chemistry including the stages of PC aminosilanization, surface activation, and automated spotting ensures direct covalent immobilization of minute quantities of proteins and peptides constituting the recognition unit of the sensor. The designed PC-based array of 13 model proteins and peptides, including secondary and primary antibodies (Abs), His-tagged and phosphorylated proteins (RAD51 and phosphorylated BSA), phosphorylated and non-phosphorylated peptide fragments of RAD51, and BSA exhibited highly specific detection of a number of model Ab analytes in the microfluidic mode within a single assay. Employing high-affinity Abs and depositing higher quantities of the capture molecules have been shown to enhance the sensor response without additional signal amplification. The limit of protein/peptide detection using the PC SM imaging approach has been calculated to be in the femtogram range, which indicates a higher sensing capacity of the engineered sensing platform compared to the conventional SPR-based techniques. Moreover, surface regeneration using an organic solvent and UV treatment makes the PC chips highly reusable. Hence, the new approach based on PC SM imaging combined with the high performance of protein immunoassays provides ultrasenstitive, quick, and low-cost detection of protein/peptide interactions.

The obtained results provide the first-time demonstration, using the PC SM technique, of the (1) simultaneous measurement of molecular interactions of a significant number of very different proteins deposited on the PC, and (2) evaluation of the optimal amount of protein for PC depositing that provides a clear signal increase when an analyte solution is introduced into the microfluidic cell. Thus, the results provide a real proof-of-concept paving the way to the future use of PC SM imaging systems as advanced tools for label-free detection of protein interactions applicable for high-throughput screening in diagnostics, drug discovery, and enzymatic kinetics applications. Moreover, the developed approach is flexible and can be further adapted for the fabrication of different protein arrays which may be used in the many fields of molecular sciences.

## Data Availability

Not applicable.

## Supplementary Materials

The following supporting information can be downloaded at: https://www.mdpi.com/1422-0067/24/5/4347/s1.
