# Supplementary material for: Label-Free Multiplexed Microfluidic Analysis of Protein Interactions Based on Photonic Crystal Surface Mode Imaging
Source: Int J Mol Sci. 2023 Feb 22;24(5):4347. doi: 10.3390/ijms24054347 (PMC10002048; doi:10.3390/ijms24054347)
Supplement: Supplementary file 1 [file ijms-24-04347-s001.zip › ijms-2182256-supplementary.pdf]

Table S1. Proteins and peptides used for engineering of a label-free photonic crystal mode imaging chip for multiplexed microfluidic analysis of protein interactions.

Sample	Description	Sample concentration before spotting, μg/mL	Approximate quantity of protein/peptide spotted (0.4 nL/drop), drop number: protein quantity
Secondary Ab-IRD800	Secondary anti-rabbit antibody conjugated with IRDye 800CW<br>0.5 mg/mL solution in phosphate-buffered saline (pH 7.4) containing 10 mg/mL BSA and 0.01% sodium azide.<br>Dilution, 1 : 2.	250	1 : 0.1ng<br>2 : 0.2 ng<br>5 : 0.5 ng<br>10 : 1.0 ng
Secondary Ab-AF680	Highly cross-adsorbed secondary anti-mouse immunoglobulin G (H+L) antibody produced in goat and conjugated with Alexa Fluor 680<br>2 mg/mL solution in 0.1 M phosphate-buffered saline (pH 7.5) containing 0.1 M NaCl and 5 mM sodium azide.<br>Dilution, 1 : 2.	1000	1 : 0.4 ng<br>2 : 0.8 ng<br>5 : 2.0 ng<br>10 : 4.0 ng
Protein A	Pierce™ immunoglobin G-binding protein A<br>5 mg/mL solution in phosphate-buffered saline.<br>Undiluted.	5000	1 : 2.0 ng<br>2 : 4.0 ng<br>5 : 10.0 ng<br>10 : 20.0 ng
Protein G	Pierce™ immunoglobin G-binding recombinant protein G<br>5 mg/mL in phosphate-buffered saline.<br>Dilution, 1 : 4.	1250	1 : 0.5 ng<br>2 : 1.0 ng<br>5 : 2.5 ng<br>10 : 5.0 ng
RAD51	DNA repair protein,<br>1.3 mg/mL solution in a buffer containing 50 mM Tris HCl (pH 7.5), 10% glycerol, 500 mM NaCl, 1 mM EDTA, 1 mM DTT.<br>Undiluted.	1300	1 : 0.5 ng<br>2 : 1.0 ng<br>5 : 2.6 ng<br>10 : 5.2 ng
pY315	KIY(P)DSPCLPEAEAMFY sequence of RAD51peptide fragment, 1 mg/mL solution in ultrapure water.<br>Undiluted.	1000	5 : 2.0 ng<br>10 : 4.0 ng
Y315	KIYDSPCLPEAEAMFY sequence of RAD51 peptide fragment,<br>1 mg/mL solution in ultrapure water.<br>Undiluted	1000	1 : 0.4 ng<br>2 : 0.8 ng
BSA	Pierce™ Bovine Serum Albumin Standard<br>2 mg/mL solution in ultrapure 0.9% saline with 0.05% sodium azide.<br>Undiluted.	2000	1 : 0.8 ng<br>2 : 1.6 ng<br>5 : 4.0 ng<br>10 : 8.0 ng
Phosphotyrosine-BSA	Bovine serum albumin phosphorylated at tyrosine residues<br>2 mg/mL solution in 0.01 M phosphate-buffered saline (pH 7.4) containing 15 mM sodium azide.<br>Undiluted.	2000	1 : 0.8 ng<br>2 : 1.6 ng<br>5 : 4.0 ng<br>10 : 8.0 ng

Anti-RAD51 Ab	Polyclonal rabbit antibody for detection of RAD51<br>0.25 mg/mL solution in 0.1 M Tris-glycine (pH 7.4) containing 0.15 M NaCl and 0.05% sodium azide.<br>Dilution, 1 : 2.	125	1 : 0.05 ng<br>2 : 0.10 ng<br>5 : 0.25 ng<br>10 : 0.50 ng
Anti-phosphotyrosine Ab	High-affinity monoclonal mouse antibody for detection of the phosphorylated forms of the peptides<br>1.5 mg/mL solution in 10 mM sodium HEPES (pH 7.5) containing 150 mM NaCl, 100 μg/mL BSA, 50% glycerol, and 0.02% sodium azide.<br>Dilution, 1 : 4.	375	1 : 0.15 ng<br>2 : 0.30 ng<br>5 : 0.75 ng<br>10 : 1.50 ng
Anti-His tag Ab	Anti-polyhistidine monoclonal antibody produced in mice<br>1 mg/mL immunoglobulin G2A in ascites fluid containing 15 mM sodium azide.<br>Dilution, 1 : 4.	250	1 : 0.10 ng<br>2 : 0.20 ng<br>5 : 0.50 ng<br>10 : 1.00 ng
Anti-pY315 Ab	Rabbit anti-pY315 antibody<br>0.02 mg/mL in Tris (pH 7.5) containing 0.02% sodium azide.<br>Dilution, 1 : 4.	5	2 : 0.004 ng<br>5 : 0.010 ng<br>10 : 0.020 ng

Table S2. Antibody analytes used in the protein assay by means of a label-free photonic crystal mode imaging chip.

Antibody analyte	Concentration of antibody in analyte solution, μg/mL
Anti-phosphotyrosine monoclonal mouse antibody	7.5
Anti-His tag monoclonal mouse antibody	20.0
Anti-rabbit polyclonal antibody	50.0

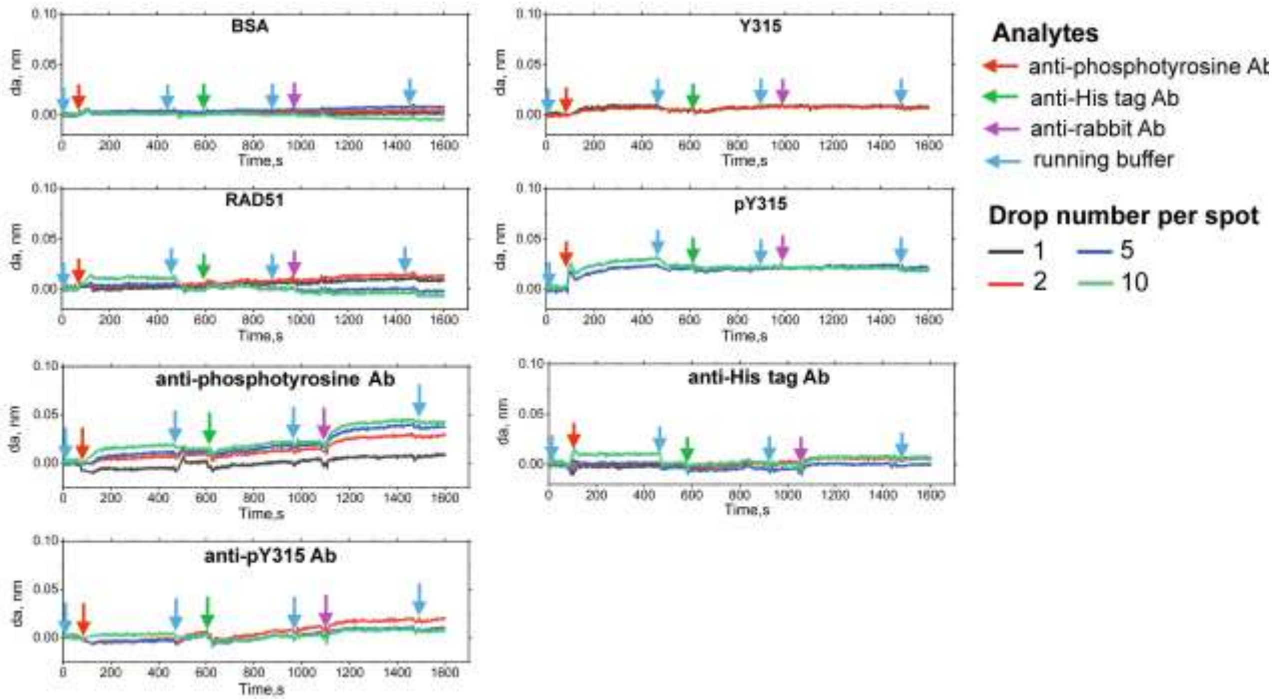

Figure S1. The sensorgrams of protein interactions. The start of the injection of each analyte run through the microfluidic cell is indicated with an arrow. The changes in adlayer thickness over BSA spots with time were considered to reflect the impact of nonspecific binding of the antibody analytes to chip surface preliminarily blocked with BSA. The resultant sensorgrams are presented as an average of 2 sensorgrams recorded for spot duplicate. Abbreviations: da, adlayer thickness; Ab, antibody; Y315, Y315 peptide; phospho-Y315, phospho-Y315 peptide; RAD51, DNA repair protein; BSA, bovine serum albumin; anti-phosphotyrosine Ab, antibody for detection of phosphorylated proteins; anti-phospho-Y315 Ab, antibody for detection of phospho-Y315 peptide; anti- His tag Ab, anti-polyhistidine antibody.

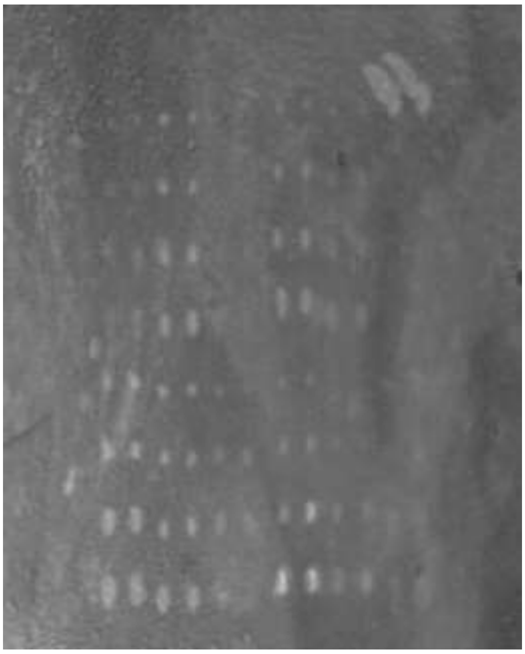

Figure S2. Black-and-white 2D image of the chip surface after antibody analyte running.

Table S3. Maximum gain of adlayer thickness over the protein/peptide spots after running of all antibody analytes.

**Protein or peptide spotted**	**Mean value of the maximum gain of adlayer thickness in spots with different protein/peptide quantities**			
	1 drop/spot	2 drops/spot	5 drops/spot	10 drops/spot
Ab-IRD800*	0.04314 ± 0.00414	0.06082 ± 0.00623	0.07969 ± 0.00843	0.11701 ± 0.00054
Ab-AF680*	0.17082 ± 0.01716	0.17600 ± 0.01940	0.20216 ± 0.02016	0.26474 ± 0.01353
Protein G*	0.08697± 0.01632	0.09692 ± 0.01418	0.11945 ± 0.01632	0.12391 ± 0.01988
Protein A*	0.06613 ± 0.00116	0.06843 ± 0.00001	0.08414 ± 0.00004	0.19865 ± 0.01874
Y315*	0.00981 ± 0.00185	0.0086 ± 0.00503	NA	NA
pY315*	NA	NA	0.02105 ± 0.00554	0.01912 ± 0.00997
RAD51*	0.00988 ± 0.00481	0.01278 ± 0.00001	-0.00208 ± 0.00357	-0.00727 ± 0.00349
phosphotyrosine BSA*	0.01405 ± 0.00121	0.05824 ± 0.00371	0.12785 ± 0.01209	0.15011 ± 0.01209
BSA (reference)	0.00197 ± 0.00151	0.00448 ± 0.00634	0.00821 ± 0.00646	-0.00436 ± 0.00617
anti-phosphotyrosine BSA Ab	0.00784 ± 0.00001	0.02914 ± 0.00380	0.03857 ± 0.00888	0.04313 ± 0.01686
anti-RAD51 Ab	-0.00010 ± 0.00001**	0.01332 ± 0.01034**	0.02058 ± 0.0082**	0.09367 ± 0.01734
anti-pY315 Ab	NA	0.02045 ± 0.00371	0.00971 ± 0.01095	0.00801 ± 0.01357
anti-His tag Ab	0.00629 ± 0.01507	0.00359 ± 0.00158	-0.00176 ± 0.00172	0.00560 ± 0.00452

* Significant differences between the mean values of the maximum adlayer thickness in protein/peptide spots compared to the reference BSA spots ($p < 0.05$, Student's *t* test). **Nonsignificant differences between the mean values of the maximum adlayer thickness in anti-RAD51 Ab and BSA spots ($p > 0.05$, Student's *t* test).
